# Paper‐Based Hydroelectric Generators for Water Evaporation‐Induced Electricity Generation

**DOI:** 10.1002/advs.202304482

**Published:** 2023-09-23

**Authors:** Jingjing Zhang, Peng Cui, Jingjing Wang, Huan Meng, Ying Ge, Can Feng, Huimin Liu, Yao Meng, Zunkang Zhou, Ningning Xuan, Bao Zhang, Gang Cheng, Zuliang Du

**Affiliations:** ^1^ School of Materials Science and Engineering Key Lab for Special Functional Materials of Ministry of Education National & Local Joint Engineering Research Center for High‐efficiency Display and Lighting Technology Collaborative Innovation Center of Nano Functional Materials and Applications Henan University Kaifeng 475004 China

**Keywords:** evaporation, hydroelectric generators, membrane, paper, power generation

## Abstract

The research presented in this paper introduces a novel environmental energy‐harvesting technology that harnesses electricity from the evaporation of water using porous structural materials. Specifically, a strategy employing paper‐based hydroelectric generators (p‐HEGs) is proposed to capture the energy produced during water evaporation and convert it into usable electricity. The p‐HEGs offer several advantages, including simplicity in fabrication, low cost, and reusability. To evaluate their effectiveness, the water evaporation‐induced electrical output performance of four different p‐HEGs are compared. Among the variants tested, the p‐HEG combining wood pulp and polyester fiber exhibits the best output performance. At room temperature, this particular p‐HEG generates a short‐circuit current and open‐circuit voltage of ≈0.4 µA and 0.3 V, respectively, thereby demonstrating excellent electrical stability. Furthermore, the electrical current and voltage generated by the p‐HEG through water evaporation are able to power an LED light, both individually and in series and parallel connections. This study delves into the potential of electricity harvesting from water evaporation and establishes it as a viable method for renewable energy applications.

## Graphical abstract

In this study, a novel approach utilizing paper‐based hydroelectric generators (p‐HEGs) is presented to harness energy from water evaporation and convert it into electricity. The underlying mechanism of p‐HEG power generation relies on the electrokinetic effect, which is induced by the pressure gradient‐induced changes in the electrical double layer (EDL), particularly the streaming potential.

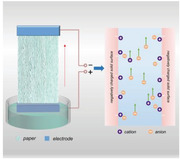

## 1 Introduction

The neglected energy in the environment can be collected and converted into electricity, which provides a new solution to the increasingly serious energy crisis.^[^ ^1^, ^2^, ^3^, ^4^, ^5^ ^]^ Water evaporation‐induced power generation is a new energy conversion method that uses water as the medium to directly convert the heat in the surrounding environment into electricity.^[^ ^6^, ^7^, ^8^ ^]^ It is natural, ubiquitous, and sustainable. The whole process does not require the input of external mechanical energy and directly produces DC output as green energy, attracting widespread attention.^[^ ^9^, ^10^ ^]^

In 2017, Guo et al. conducted a study revealing that carbon black with a centimeter‐level porous structure has the capability to generate volt‐level voltage through water evaporation. However, the high resistance of the carbon black sheet limits the current flow.^[^ ^11^ ^]^ Since then, researchers have made advancements in improving power generation performance by modifying various factors including material composition, microstructure, surface functional group density, and device fabrication process.^[^ ^12^, ^13^, ^14^ ^]^ Other carbon‐based materials such as carbon nanotubes, graphene oxide (GO), and graphene have also been explored for evaporation‐induced power generation due to their hydrophilicity, large surface area, and surface charging properties.^[^ ^15^, ^16^, ^17^ ^]^ Nonetheless, there exists a need to investigate alternative materials for hydroelectric generators. Metal oxides with a high zeta potential, for instance, have shown promise in driving evaporation‐based power generation. Qu et al. demonstrated the generation of 2.5 V voltage by coating Al_2_O_3_ on PET to create a hydroelectric generator.^[^ ^18^ ^]^ However, the disorderly microscopic pore structure in such systems can interfere with ion movement in the solution, impacting power output. To address this, Sun et al. proposed the use of a hydroelectric device incorporating a silicon nanowire array with an ordered aligned structure, resulting in a power density of 6 µW cm^−2^.^[^ ^19^ ^]^ Composite materials like carbon nanohemispheres and TiO_2_ nanowires have also been suggested, where the super‐hydrophilic surface of TiO_2_ nanowires combined with carbon materials exhibited a voltage of 1.6 V and significantly improved power output.^[^ ^20^ ^]^ Despite progress in evaporation‐induced power generation, challenges such as limited power output, complex preparation processes, and high costs hinder its wide‐scale application.^[^ ^21^ ^]^

In contrast, cellulose‐based paper offers a range of advantages including low cost, flexibility, self‐supporting properties, environmental friendliness, and abundant oxygen‐containing functional groups.^[^ ^22^ ^]^ This type of paper is commonly composed of plant fibers and various fillers, and it possesses a porous structure that facilitates the directional movement of water during evaporation. Here, we propose the utilization of paper‐based hydroelectric generators (p‐HEGs) for power generation driven by water evaporation, which demonstrates simplicity, cost‐effectiveness, and reusability. We compared the power generation performance of four different p‐HEGs made of commercial paper. Notably, the paper consisting of wood pulp and polyester fiber exhibited superior hydrophilicity and achieved the highest output, generating a short‐circuit current of ≈0.42 µA and an open‐circuit voltage of 0.33 V. By connecting multiple p‐HEGs in series and parallel configurations, the current and voltage induced by evaporation were effectively enhanced, enabling the power supply for electronic devices such as LEDs. This highlights the potential application of p‐HEGs for electricity generation through water evaporation.

## 2 Results and Discussion

**Figure 1:**
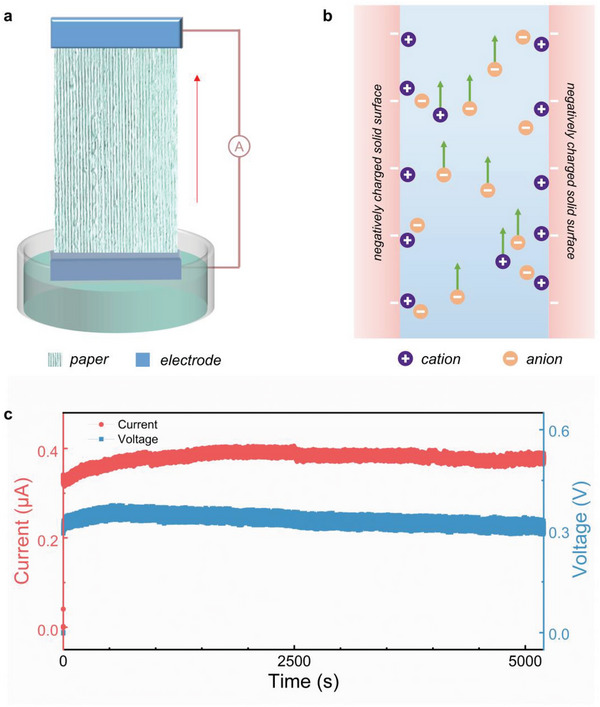
a) Schematic diagram of paper‐based HEGs used in water evaporation‐induced power generation devices. b) Schematic diagram of the water evaporation‐induced electricity generation mechanism of paper‐based HEGs. c) Output performance of composite paper‐based HEG for 1 h.

A schematic diagram of the water evaporation‐induced power generation with a p‐HEG is shown in **Figure** **1**a. The free‐standing p‐HEG is leaning against the edge of the glass dish, the upper and lower electrodes are connected to the ammeter, the bottom electrode is immersed in water, and the top electrode is exposed to air, which continuously flows from the bottom electrode to the top electrode driven by capillary forces induced by evaporation. The fabrication process of the p‐HEG is facile. The paper is commercially available, and the electrodes are used with painted silver paste. A composite paper‐based p‐HEG could generate a current of 0.38 µA and a voltage of 0.3 V lasting for >1 h induced by water evaporation, as shown in (Figure 1c). Furthermore, we have demonstrated that the p‐HEG exhibits excellent stability, as it continues to generate output even after repeated usage and storage under ambient conditions for >10 months. This finding is illustrated in Figure S1 (Supporting Information). The long‐term stability of the p‐HEG further highlights its potential for practical applications in energy harvesting.

The principle of electricity generation is described in Figure 1b. Initially, when a liquid comes into contact with a charged surface, an electrical double layer (EDL) is formed, primarily comprised of the Stern layer and the diffusion layer.^[^ ^23^, ^24^, ^25^ ^]^ According to the Gouy–Chapman–Stern model, the Helmholtz layer generates a fixed capacitance, C_H_, while the diffusion layer generates a capacitance, C_D_. The relationship between these two capacitances and the double‐layer capacitance, C_d_, can be described as follows.^[^ ^26^ ^]^

(1) $$\frac{1}{C_{d}}=\frac{1}{C_{H}}+\frac{1}{C_{D}}$$

Under certain pressure gradients, a relative displacement occurs between the solid and liquid, resulting in dynamic changes in the EDL. This gives rise to the classical electrokinetic effects based on the EDL, particularly the streaming potential, which involves the movement of ionic solutions through narrow gaps.^[^ ^27^, ^28^ ^]^

Upon absorbing water, p‐HEG undergoes ionization of the oxygen‐containing functional groups present on the cellulose fibers and fillers, resulting in the acquisition of negative charges. Consequently, the positive ions within the water adhere to the surface of the fibers, thus forming an electrical double layer.^[^ ^29^, ^30^ ^]^ Driven by evaporation or capillary force, the negative ions migrate through the material and accumulate at the evaporation surface. Conversely, positive ions move toward the opposite side, generating a potential difference. This potential difference induces the flow of current once p‐HEG is connected to an external circuit.

The aforementioned mechanism is derived from theoretical analysis and is supported by the streaming potential/current equation, as well as the equations related to capillary pressure and hydrodynamic flow resistance.^[^ ^31^ ^]^ The remaining contact angle (*θ*), zeta potential (*ζ*), and aperture (*d*) serve as essential parameters for comparing the four membrane papers, collectively influencing the streaming current/potential (Table S1, Supporting Information).^[^ ^32^, ^33^ ^]^

**Figure 2:**
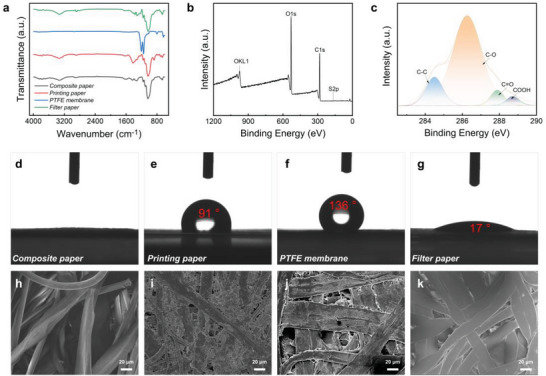
a) FTIR spectra of the four kinds of paper. b,c) XPS spectrum of paper composed of wood pulp and polyester fiber. d–g) Contact angle of a 5 µL droplet on the four papers for the same time. h–k) SEM images of the four different papers.

Composite paper, printing paper, PTFE membrane, and filter paper are selected as the materials for construction of the p‐HEGs, which are composed of wood pulp and polyester fibers, plant fibers and fillers, polytetrafluoroethylene and cotton fibers, respectively. **Figure** **2**a shows the FT‐IR spectra of the four papers, among which the papers with wood pulp and polyester fiber have peaks (black lines) at 1034, 1206, 1593, and 3338 cm^−1^, indicating that these papers contain ester, carbonyl, hydroxyl, and other oxygen‐containing groups. The peaks of the printing paper (red line) at 1031, 1420, 1724, and 3701 cm^−1^ and those of the filter paper (green line) at 1031, 1102, and 1158 cm^−1^ indicate that they contain carbonyl, hydroxyl, ether, epoxy, and other oxygen‐containing groups, respectively. In addition, the stretching vibration and anti‐stretching vibration peaks of the C─F bond are observed at 1148 and 1205 cm^−1^ for the PTFE membrane paper (blue line). The four kinds of papers are also characterized by XPS, and the XPS results of the paper with wood pulp and polyester fiber are shown in (Figure 2b,c). The oxygen‐containing groups mainly include C─C, C─O, C═O, and COOH, as indicated by the peaks at 284.5, 286.3, 288, and 288.6 eV. The printing paper and filter paper also show the above four peaks, and their surfaces also show signals for the above oxygen‐containing functional groups (Figures S2 and S3, Supporting Information). However, in addition to showing the C─O bond signal at 286.4 eV, the peak surface of the PTFE filtration membrane also shows C─F bond signals at 292.2 and 293.4 eV (Figure S4, Supporting Information). These oxygen‐containing groups contribute significantly to the generation of a negative charge on the surface of paper and the subsequent generation of voltage.^[^ ^34^ ^]^ After 5 microliter droplets are dropped on the four different papers for the same time, contact angles of 0°, 91°, 136°, and 17° are obtained, as shown in Figure 2d–g, indicating that the composite paper and filter paper have better hydrophilicity. As seen from the SEM diagram in Figure 2h–k, composite paper and filter paper have larger pores. The negative zeta potential of these four types of paper is shown in Figure S5 (Supporting Information), in which PTFE film paper has the highest zeta potential, followed by printer paper, and composite and filter paper have the lowest zeta potential. The result of the combined action of these factors is that the composite paper base HEG produces the highest electrical output.

**Figure 3:**
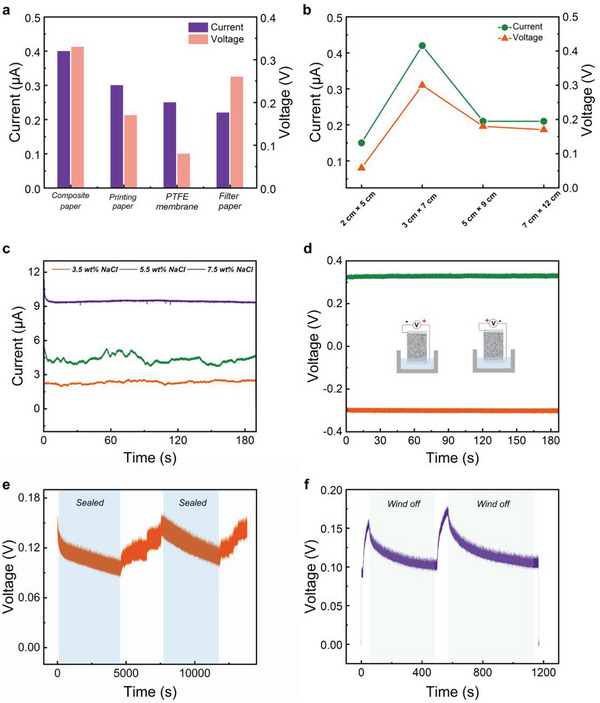
a) Comparison of the current and voltage induced by water evaporation of the four p‐ HEGs with the same size. b) Comparison of the current and voltage induced by water evaporation of the composite paper (wood pulp and polyester fiber) HEG with different areas. c) Current generated by the evaporation of sodium chloride solutions of different mass fractions. d) Changing of the connection mode between the p‐HEG and ammeter generating equal voltage with opposite polarity. e) Voltage changes resulting from PE film sealing and unsealing of the evaporation‐induced power generation unit. f) Voltage changing due to accelerated evaporation caused by blowing on the equipment.

The water evaporation‐induced electrical outputs of the four different p‐HEGs are also compared, as shown in **Figure** **3**a. The electrical output of the composite paper composed of wood pulp and polyester fiber is the largest, reaching a 0.42 µA short‐circuit current and 0.33 V open‐circuit voltage. It is cut into four p‐HEGs with different areas of 2 cm × 5 cm, 3 cm × 7 cm, 5 cm × 9 cm, and 7 cm × 12 cm for water evaporation‐induced power generation, as shown in Figure 3b, and the p‐HEG with an area of 3 cm × 7 cm has the largest electrical output. When the area is larger, the water rises slowly, and part of the water evaporates before it reaches the top of the electrode, thus affecting the electrical output. Figure 3c shows that when deionized water is substituted for a sodium chloride solution for water evaporation‐induced electricity generation, the current generated increases from 2.5 to 9.2 µA when the mass fraction increases from 3.5 to 7.5 wt.%, which highlights the important contribution of ion mobility to electricity generation.^[^ ^35^, ^36^ ^]^ In addition, to verify that the electricity generated is related to water evaporation, we perform a series of verification tests. First, the p‐HEG is placed in a waterless container and connected to an ammeter to test that no current or voltage is generated. Then, the paper in the water evaporation‐induced power generation device is removed, and no current or voltage is generated, indicating that electricity induced by water evaporation is not a primary battery effect (Figure S6, Supporting Information).^[^ ^37^ ^]^ Then, after switching the connection modes between the upper and lower electrodes of the device and the ammeter, a voltage of equal magnitude and opposite polarity is obtained, as shown in Figure 3d. In addition, the whole evaporation‐induced power generation device is placed in the beaker and sealed with PE film. The voltage generated gradually dropped, and the voltage recovered slowly after the film was unsealed, as shown in Figure 3e. This is because after the film is sealed, the humidity in the beaker increases to 100%, and no evaporation occurs. When evaporation is promoted by blowing air or heating above the p‐HEG, the current and voltage induced by water evaporation increase (Figure 3f; Figures S7 and S8, Supporting Information). When the blowing air or heating is stopped, the current and voltage slowly return to their original values. The above real experimental results show that the electricity generated is closely related to evaporation.

**Figure 4:**
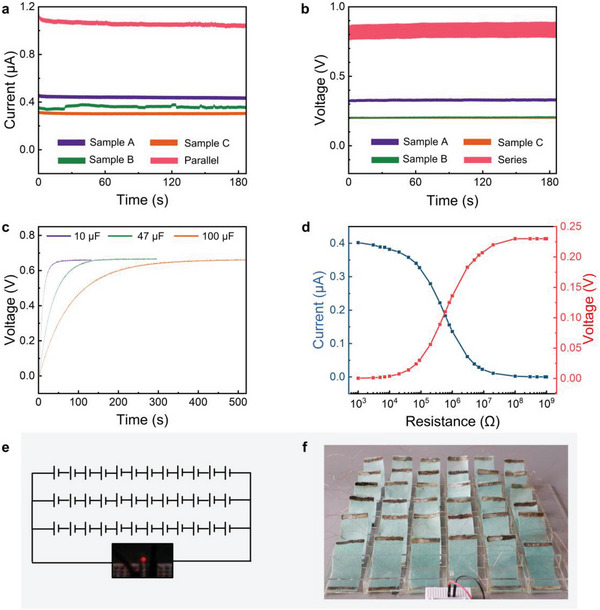
a,b) The current and voltage generated by water evaporation of three separate devices and the current and voltage generated by series or parallel connection of three devices, respectively. c) Three devices connected in series to charge 10, 47, and 100 µF capacitors. d) Changes in the current and voltage generated by the device under loads with different resistance values. e,f) Thirty‐six devices connected in series and parallel to supply power to LED lights.

The output of the device can be easily scaled by connecting multiple p‐HEGs. Connecting three devices in parallel produces a current of 1.1 µA, as shown in (**Figure** **4**a), which is three times the output of a single device. The same result is obtained when three devices are connected in series to test the voltage, as shown in Figure 4b, which produces a voltage of 0.85 V. By connecting three devices in series, commercial capacitors of 10, 47, and 100 µF can be charged to 0.67 V, as shown in Figure 4c. Figure 4d shows that the current decreases from 0.4 to 0 µA, and the voltage increases from 0 to 0.23 V as the resistance increases from 1000 to 1 GΩ. Furthermore, with 36 devices connected in series and in parallel, the current and voltage induced by water evaporation can be scaled to power a commercial LED, as shown in Figure 4e,f, which indicates the potential of our device to capture the energy from water evaporation and turn it into electricity.

**Figure 5:**
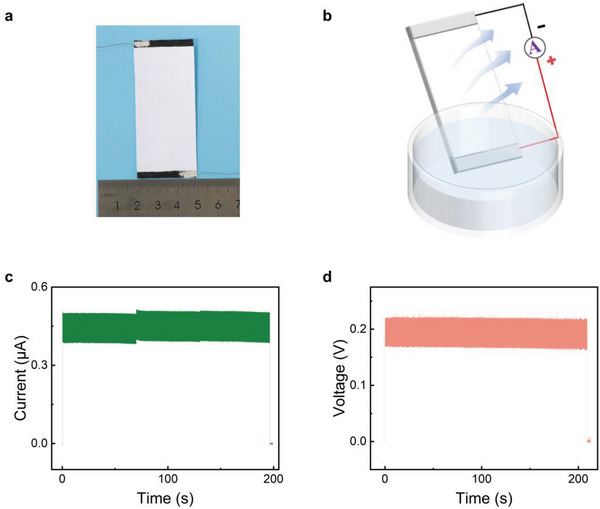
a) Optical image of a p‐HEG using carbon as electrode. b) Schematic diagram of the water evaporation‐induced power generation of p‐HEGs using carbon as the electrode. c,d) Electrical output of p‐HEG with a carbon electrode induced by water evaporation.

The electrodes on both ends of the p‐HEG are replaced with carbon electrodes, that is, graphite is applied to the upper and lower ends of the film paper with a pencil with a width of 3 mm, and then the ammeter is connected with copper wire, as shown in (**Figure** **5**a,b). The p‐HEG with a carbon electrode generates a short‐circuit current of 0.45 µA and an open‐circuit voltage of 0.2 V by water evaporation, as shown in Figure 5c,d. The raw materials for this process are paper and pencil, in other words, we can complete the collection of electric energy induced by water evaporation with a piece of paper and a pencil. Undoubtedly, this provides a green, environmentally friendly, and sustainable energy‐conversion strategy.

## 3 Conclusion

In summary, we propose a strategy utilizing p‐HEGs, which can be easily fabricated using basic materials like paper and pencil, to capture and convert electrical energy generated from water evaporation. These devices offer several advantageous features, including simplicity in production, energy conservation, environmental friendliness, and sustainability. By treating the p‐HEG with plasma, we achieve a short‐circuit current of ≈0.45 µA and an open circuit voltage of 0.2 V through water evaporation at room temperature. Furthermore, by employing silver glue as the electrode, we achieve a short‐circuit current of ≈0.42 µA and an open circuit voltage of 0.33 V. By connecting multiple devices in series and parallel, we are able to power an LED light and charge commercial capacitors. Our findings highlight the considerable potential of p‐HEGs in harnessing water evaporation for clean and renewable energy generation, which is of significant importance in promoting sustainable and environmentally friendly energy utilization.

## 4 Experimental Section

### Preparation of Paper‐Based Hydroelectric Generators

The membranes and paper used in this work are commercially available. Silver paste was chosen as the electrode material for fabrication of the devices. The electrode was connected using copper wire, and the device was allowed to dry naturally in ambient air at room temperature for further usage.

### Characterization of Paper‐Based Hydroelectric Generators

The morphologies of the p‐HEG were characterized by scanning electron microscopy (JEOL JSM‐7610F Plus). The FT‐IR spectra of the p‐HEG were characterized by a Fourier‐transform infrared spectrometer (VERTEX 70). Static contact angle tests were performed by an optical contact angle measuring system (LAUDA OSA 100). The XPS spectra of the p‐HEG were characterized by AXIS SUPRA+. The zeta potentials of different samples were collected by Anton Paar surpass. The short‐circuit currents and voltages of the HEGs were measured by an electrometer (Keithley 6514).

## Conflict of Interest

The authors declare no conflict of interest.

## Supporting information

Supporting Information

## Data Availability

The data that support the findings of this study are available from the corresponding author upon reasonable request.
