# Supplementary material for: Paper‐Based Hydroelectric Generators for Water Evaporation‐Induced Electricity Generation
Source: Adv Sci (Weinh). 2023 Sep 23;10(31):2304482. doi: 10.1002/advs.202304482 (PMC10625126; doi:10.1002/advs.202304482)
Supplement: Supplementary file 1 — Supporting Information [file ADVS-10-2304482-s001.pdf]

ADVANCED SCIENCE
Open Access

Supporting Information

for *Adv. Sci.*, DOI 10.1002/advs.202304482

Paper-Based Hydroelectric Generators for Water Evaporation-Induced Electricity Generation

Jingjing Zhang, Peng Cui, Jingjing Wang, Huan Meng, Ying Ge, Can Feng, Huimin Liu, Yao Meng, Zunkang Zhou, Ningning Xuan, Bao Zhang, Gang Cheng* and Zuliang Du*

Supplementary Information

Paper-based Hydroelectric Generators for Harvesting Water-Evaporation-Induced Electricity

Jingjing Zhang, Peng Cui, Jingjing Wang, Huan Meng, Ying Ge, Can Feng, Huimin Liu, Yao Meng, Zunkang Zhou, Ningning Xuan, Bao Zhang, Gang Cheng*, Zuliang Du*

School of Materials Science and Engineering, Key Lab for Special Functional Materials of Ministry of Education, National & Local Joint Engineering Research Center for High-efficiency Display and Lighting Technology, Collaborative Innovation Center of Nano Functional Materials and Applications, Henan University, Kaifeng, 475004, China

Email: cuipeng@henu.edu.cn; chenggang@henu.edu.cn

Discussion of the relationship between the streaming potential/current, capillary pressure and hydrodynamic flow resistance equation.

Imagine a capillary tube with length L, pore cross-sectional area A, and zeta potential ζ filled with water with pressure difference ΔP, and the streaming potential and current are expressed as [1]

$$Vs = \frac{\varepsilon_0 \varepsilon_r \Delta P \zeta}{\sigma \mu}$$

$$Is = \frac{A \varepsilon_0 \varepsilon_r \Delta P \zeta}{L \mu} \quad (1)$$

where ε_r、 ε_0, σ, and μ are the dielectric constant, conductivity and viscosity of the solution. The capillary tube cross-sectional area A = $\pi d^2/4$, where d is the aperture, and the pressure difference between the upper and lower sides is $\Delta P = 4\gamma \cos \theta / d$, where θ is the contact angle, γ is the surface tension of the water. [2] Substitute these two values into the equations 1 to get

$$Vs = \frac{4 \varepsilon_0 \varepsilon_r \gamma \zeta \cos \theta}{\sigma \mu d}$$

$$Is = \frac{\pi \gamma d \varepsilon_0 \varepsilon_r \zeta \cos \theta}{L \mu} \quad (2)$$

For the deionized water we use, ε_r、 ε_0, σ, γ, μ and L can be regarded as constants, so the remaining θ, ζ , d are important references for the comparison of the four membrane papers.

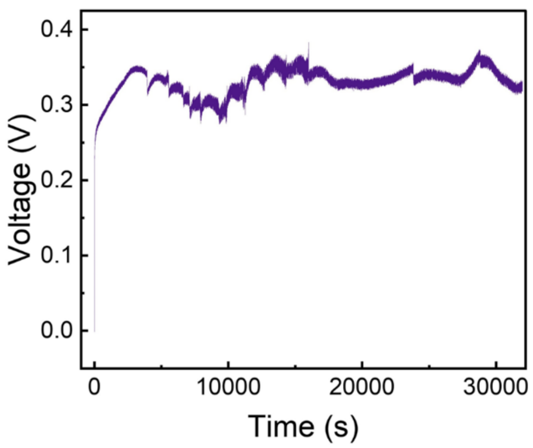

Figure S1. Stability tests of p-HEG (composite paper) which has been fabricated for 10 months.

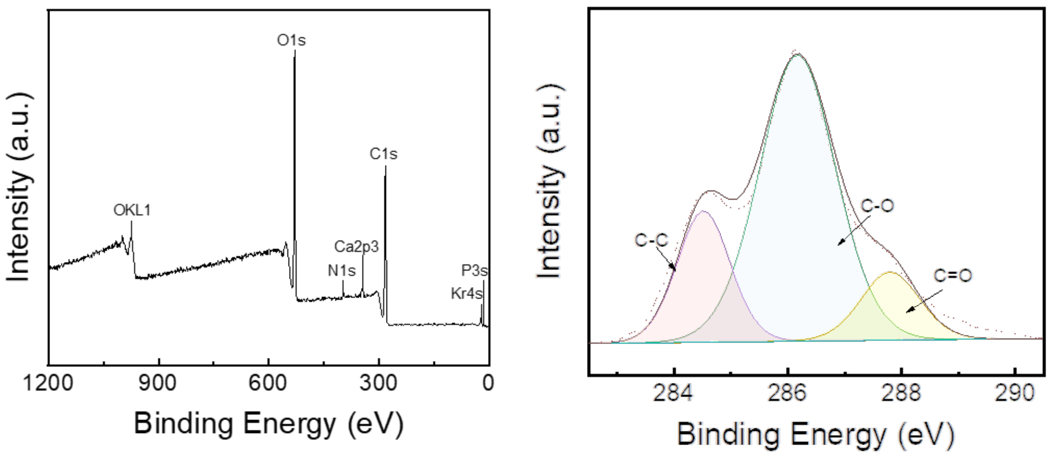

Figure S2. XPS spectra of a piece of printing paper.

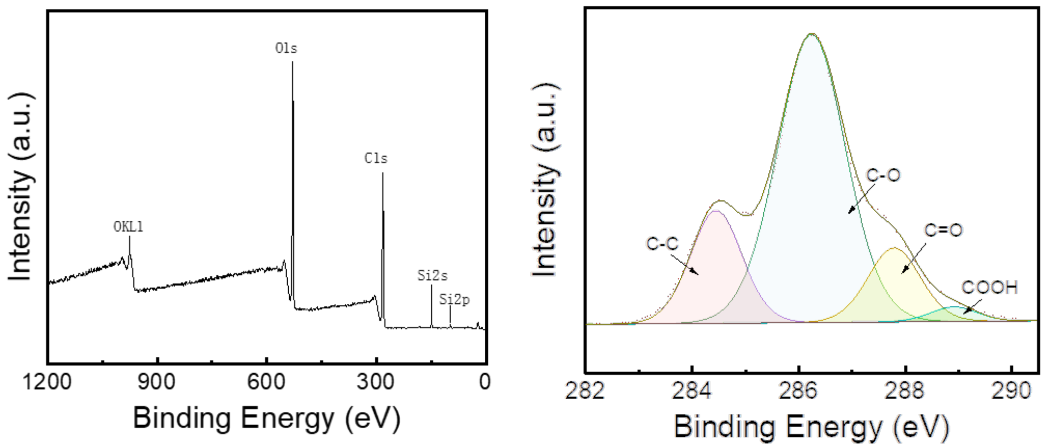

Figure S3. XPS spectra of a piece of filter paper.

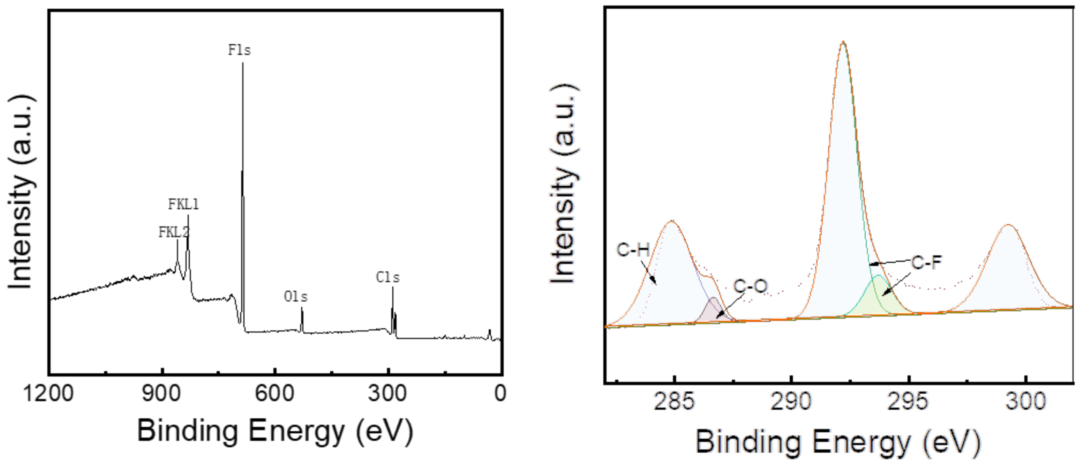

Figure S4. XPS spectra of a PTFE membrane filter.

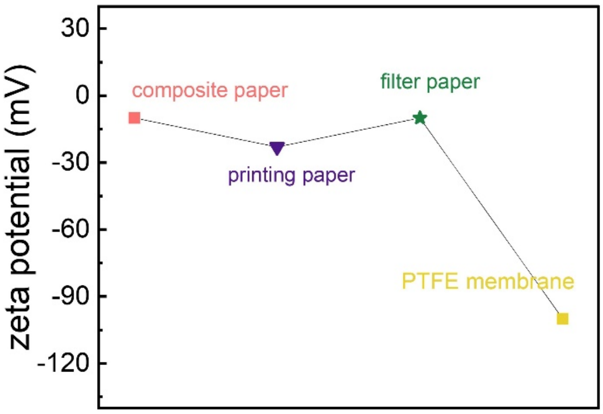

Figure S5. The zeta potentials of four kinds of samples.

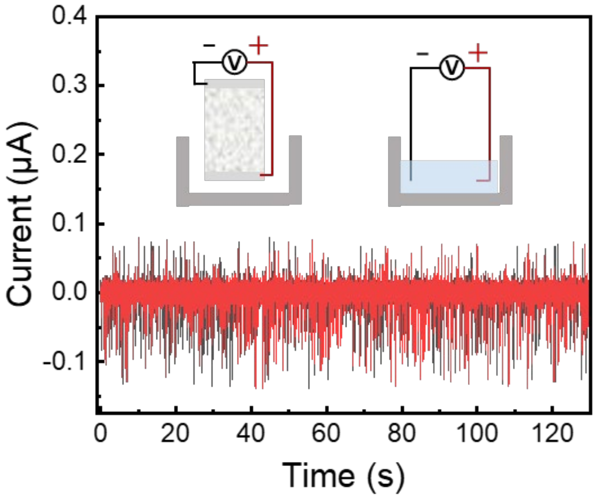

Figure S6. The electrical outputs of the composite paper HEG placed into an anhydrous container and the composite paper removed from the water evaporation induced power generation device.

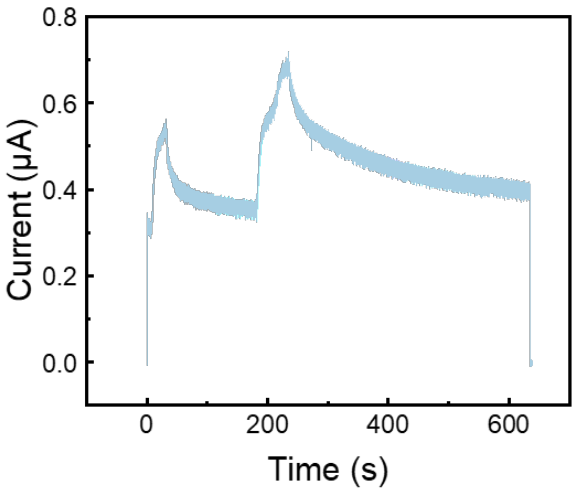

Figure S7. Current changes induced by evaporation when blowing over p-HEG.

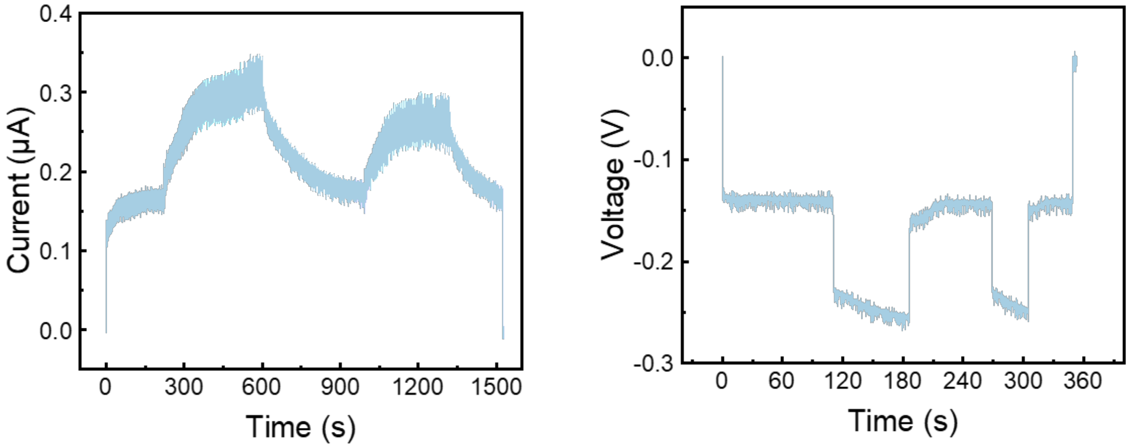

Figure S8. Changes of current and voltage induced by evaporation when heating lamp above the p-HEG is irradiated.

Table R1. Summary of parameters of four different film papers

	A (Area of p-HEG)	Θ (Contact Angle)	ζ (zeta potential)	d (aperture)
Composite paper	3×7 cm	0°	-10 mV	30.83 μm
filter paper	3×7 cm	17°	-10 mV	24.52 μm
printing paper	3×7 cm	91°	-23 mV	1.42 μm
PTFE membrane	3×7 cm	136°	-100 mV	14.3 μm

References:

[1] W. Olthuis, B. Schippers, J. Eijkel, A. van den Berg, *Sens. Actuators*, *B* **2005**, 111−112, 385−389.

[2] P.-G. d. Gennes, F. Brochard-Wyart, D.´ Queré, *Physics Today* **2004**, 57, 66–67.
